# Cleidocranial Dysplasia Case Report: Remodeling of Teeth as Aesthetic Restorative Treatment

**DOI:** 10.1155/2014/901071

**Published:** 2014-06-18

**Authors:** Leonardo Fernandes da Cunha, Isabela Maria Caetano, Fernando Dalitz, Carla Castiglia Gonzaga, José Mondelli

**Affiliations:** ^1^Graduate Program in Dentistry, Positivo University, 5300 Rua Professor Pedro Viriato Parigot de Souza, 81280-330 Curitiba, PR, Brazil; ^2^Department of Orthodontics, Hospital for Rehabilitation of Craniofacial Anomalies, University of São Paulo, Al. Octávio Pinheiro Brisolla 9-75, Vila Universitária, 17012-901 Bauru, SP, Brazil; ^3^University of São Paulo, Bauru, Al. Octávio Pinheiro Brisolla 9-75, Vila Universitária, 17012-901 Bauru, SP, Brazil

## Abstract

Cleidocranial dysplasia (CCD), is an autosomal dominant disorder with a prevalence of 1 in 1,000,000 individuals. It is generally characterized by orofacial manifestations, including enamel hypoplasia, retained primary teeth, and impacted permanent and supernumerary teeth. The successful treatment involving a timing intervention (orthodontic-maxillofacial surgeons-restorative) is already described. However, the restorative treatment might improve the aesthetic final result in dentistry management for patients with cleidocranial dysplasia. *Objective*. Therefore, this clinical report presents a conservative restorative management (enamel microabrasion, dental bleaching, and direct composite resin) for aesthetic solution for a patient with CCD. *Clinical Considerations*. The cosmetic remodeling is a conservative, secure, and low cost therapy that can be associated with other procedures such as enamel microabrasion and dental bleaching to achieve optimal outcome. Additionally, the Golden Proportion can be used to guide dental remodeling to improve the harmony of the smile and the facial composition. *Conclusions*. Thus, dentists must know and be able to treat dental aesthetic problems in cleidocranial dysplasia patients. The intention of this paper is to describe a restorative approach with the cosmetic remodeling teeth (by grinding or addicting material) associated with enamel microabrasion and dental bleaching to reestablish the form, shape, and color of smile for patients with cleidocranial dysplasia.

## 1. Introduction

Cleidocranial dysplasia (CCD) is a rare (1 : 1.000.000) autosomal dominant inheritance skeletal syndrome related to numerous dental abnormalities such as delayed eruption, retention of the permanent dentition, and highly arched palate [1]. Multidisciplinary cooperation between orthodontists and oral and maxillofacial surgeons is previously described in the literature for treatment of the CCD [2–5]. Nevertheless, in some situations, the orthodontic and maxillofacial surgeons' intervention is not sufficient to meet the patient's smile aesthetic expectations due to, for example, the presence of enamel hypoplasia or supernumerary teeth in dental arch. In these circumstances, restorative intervention probably improves the final results.

Enamel hypoplasia can be thoroughly treated by the abrasive action of a microabrasion with pumice and acid solutions as described by Croll and Cavanaugh [6, 7]. In addition, bleaching is a conservative procedure routinely used. The association of these techniques is possible and a particularly interesting option to other more invasive aesthetic procedures in esthetic improvement of patients with enamel hypoplasia and/or CCD.

Besides, when supernumerary teeth and dental anomalies of size and/or shape are present, other aesthetic treatments are required. In such situations, remodeling of teeth by grinding can be considered a safe procedure to contour a tooth surface or as an adjunct procedure during orthodontic or restorative treatments [8–11]. It is a conservative method since tooth reduction can be controlled and adequate improvement in the aesthetics and function is produced immediately with low cost.

The grinding process must not be done without restorative planning. The Golden Proportion indicated by Levin and Lombardi has been suggested as one potential mathematic method to transmit harmonious dental composition [12, 13]. This concept of proportion may be used to assist the remodeling intervention in developing esthetically beautiful smile.

The remodeling of teeth is also done by adding restorative material. The adhesive system and layering technique with composite resin can enhance better anatomic form, shape, and color [14] with minimal invasive procedures. With the improvement of the adhesive restorative materials, the successful aesthetic and stability result of this technique has been advantaged.

Cleidocranial dysplasia is a rare syndrome and these patients are seeking to improve their dental appearance. Dentists must be able to solve these situations and an interdisciplinary approach can be an interesting option for achieving predictable outcomes. Hence, the intention of this paper is to describe an association of conservative procedures (enamel microabrasion, dental bleaching, and remodeling of teeth) as restorative solution for an anterior dental composition of a patient with cleidocranial dysplasia after cooperation involving orthodontists and oral and maxillofacial surgeons.

## 2. Case Presentation

**Figure 1:**
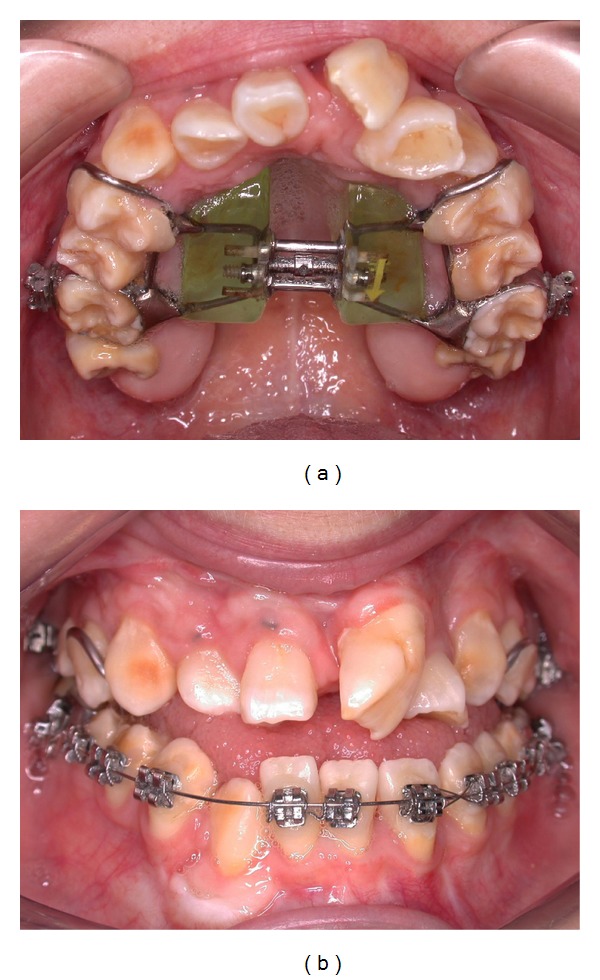
Preoperative view of patient's smile with cleidocranial dysplasia before orthodontic treatment.

**Figure 2:**
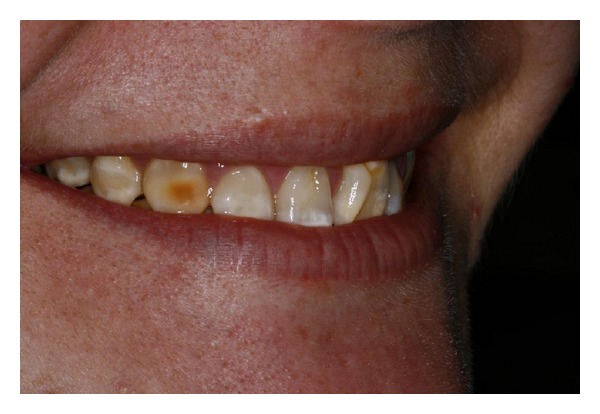
Preoperative view of patient's smile with cleidocranial dysplasia after orthodontic treatment.

**Figure 3:**
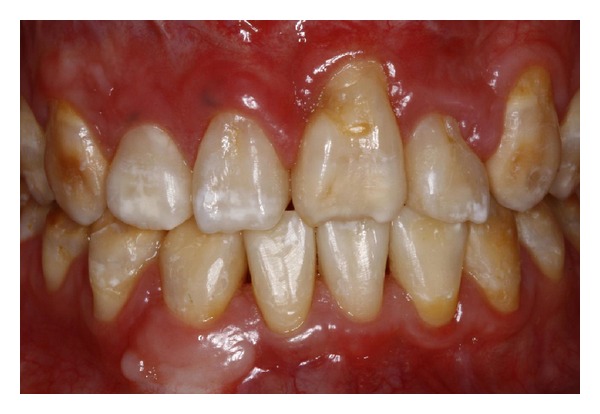
Close-up view of the anterior teeth after orthodontic treatment. Note the compromised aesthetics due to enamel hypoplasia and anatomic discrepancies of form, shape, and color.

Female patient, 25 years old, with cleidocranial dysplasia, presented for treatment to the Department of Restorative Dentistry, Bauru School of Dentistry, University of São Paulo, Brazil. Medical history revealed that orthodontics and maxillofacial surgery were previously involved (Figures 1(a) and 1(b)). Radiographic images and clinical exam were made. Clinically, enamel hipoplasia, different shape, and form were observed in all anterior dental composition. In addition, the gingival contouring was evaluated and considered to be not reproducing the harmonious architecture (Figures 2 and 3). However, no periodontal surgery was done because the gingival margin does not become visible in her smile. This was discussed with the patient and her wish was respected.

**Figure 4:**
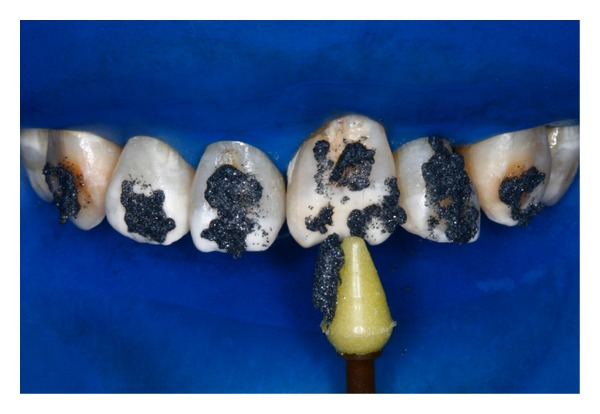
After rubber dam placement, application of the microabrasive product on the surface of the stained enamel with intermittent appliance.

Initially, microabrasion technique was executed. Glasses were used to protect the patient's and professional's eyes during the operative process, even as rubber dam, to prevent contact of the product with the gingival tissue. The microabrasive product (Whiteness RM, FGM Produtos Odontológicos Ltda., Joinville, Brazil) was applied on the enamel with surface irregularities or stains, following the manufacturers' instructions. And so with the aid of a synthetic rubber and gear reduction angle superficial enamel discoloration was removed after two applications (Figure 4). Water spray was applied between each application.

Succeeding, immediate bleach technique was performed with hydrogen peroxide (Pola Office +, SDI, Victoria, Australia), following the manufacturers' instructions. The bleaching agent was applied three times on superior and inferior anterior teeth.

**Figure 5:**
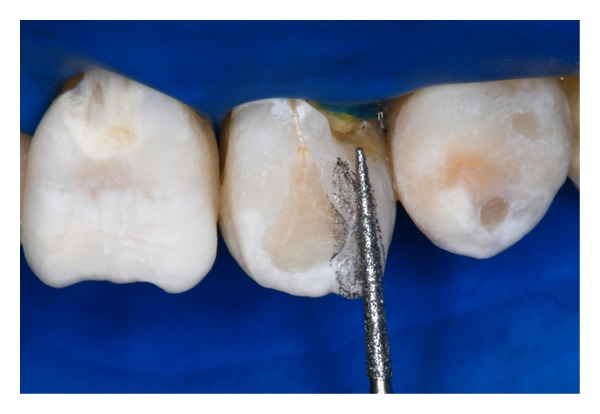
After application of hydrogen peroxide gel on the surface of the anterior teeth in agreement with the immediate bleaching procedure, remodeling by grinding of the supernumerary enamel's surface with 3203# bur.

After the microabrasion technique and bleaching treatment, a remodeling by grinding was performed at the buccal face of the supernumerary teeth (Figure 5) and left central incisor. The ground enamel surfaces were then polished with OptiDisc (Super-Tray, Kerr, Joinville, SC, Brazil).

**Figure 6:**
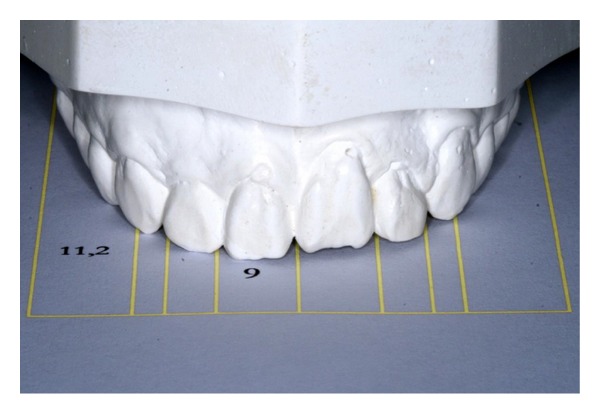
Dental model for remodeling planning according to “the Golden Proportion.”

As a restorative planning, before the remodeling by adding restorative material, the quantity of space and material needed was evaluated. The restorative planning with supplementary grinding and the addition of material was executed in accordance with the Golden Proportion model (Figure 6).

**Figure 7:**
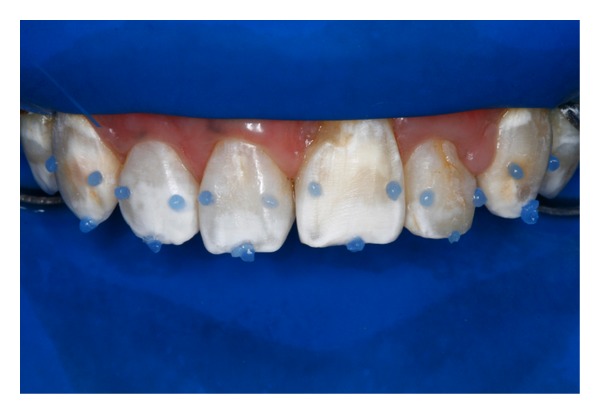
Simplified technique for rubber dam placement and acid etching at restricted points of the enamel surface was performed to diagnostic mock-up and tissue conditioning.

**Figure 8:**
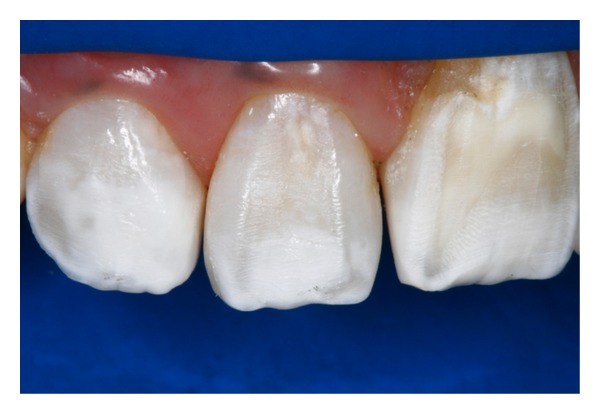
Following the grinding of the upper left central incisor with diamond disk to improve symmetry across the midline and tooth discrepancies.

**Figure 9:**
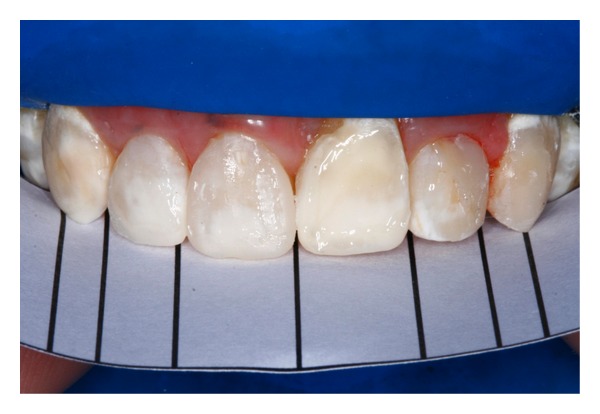
Mock-up with composite resin.

In a subsequent session, according to the restorative planning, further remodeling by grinding was performed. Simplified technique for rubber dam placement was used [15]. The enamel was removed from the distal surface of the lateral incisors and canines to improve the recurring dental proportion proceeding distally in the arch [13]. Interproximal enamel reduction was indicated with diamond bur disk (Mani, Kiohara, Japan) in a contra-angle handpiece for elimination of tooth-size discrepancy between central incisors. A mock-up restoration to more accurately define color and shape was previously done before the cosmetic remodeling by addition of material. An acid etching was performed at restricted points of the enamel surface [16] (Figures 7, 8, and 9).

Cosmetic remodeling by adding restorative material of the upper anterior teeth was performed by addition of resin composite. An etch-and-rinse adhesive system was applied according to the manufacturer's instructions (OptiBond FL, Kerr). A nanohybrid resin composite was used (Premisa, Kerr, Brazil). Addition of material was applied to reestablish the midline of the central incisors, to correct the morphologic asymmetry of the supernumerary tooth, and on the upper canines to improve color between the anterior teeth. A thin layer of dentin shade (A2) was firstly inserted to simulate the opacity of the dentine, followed by enamel shade (A2). An LED curing light was used (Radii-cal, SDI).

**Figure 10:**
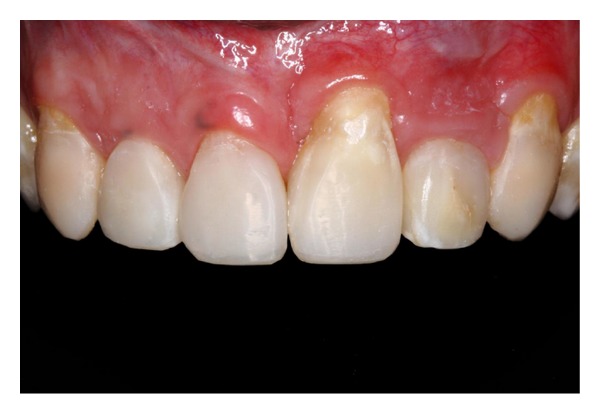
Close-up view of the anterior teeth after finishing and polishing the direct adhesive restorations.

**Figure 11:**
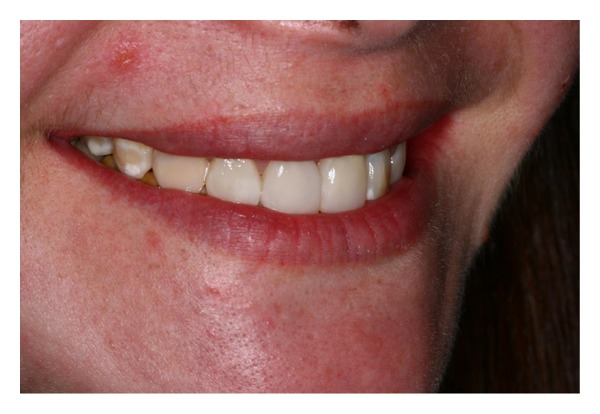
The harmony of the dental composition and the smile was reestablished after remodeling management.

The final restorative phase was achieved by contouring and finishing the restorations using laminated burs and sequential discs. The polishing was accomplished with composite polishing paste (Diamond Polishing Paste, Kerr/Sybron, CA). Final restorations can be observed in Figures 10 and 11.

## 3. Discussion 

The treatment of cleidocranial dysplasia requires multidisciplinary intervention [2–5]. A restorative approach has an imperative role in the final outcome of the treatment because the existence of enamel hypoplasia or supernumerary teeth may disturb the harmony of the smile [12]. In the case presented, either direct resin composite or indirect porcelain veneers could be performed. However a more conservative management can be indicated preceding more invasive therapies.

Microabrasion and dental bleaching can be considered a secure treatment and, furthermore, a conservative alternative [17, 18]. Opportunely these treatments can be associated with other therapies.

Dental remodeling was the conduct selected due the advantages offered by this technique. It can be done in a single session, thus contributing to the lower cost of this procedure when compared to porcelain laminate veneers for example. Even extensive recontouring by grinding is also secure, without discomfort or significant pulp and dentin reactions [9, 10].

According to Snow [19], symmetry across the midline, anterior or central dominance, and regressive proportion are three composition elements required to create esthetics in a smile. The symmetry across the midline guided the interproximal enamel reduction of the left central incisor to the elimination of tooth-size discrepancies between upper central incisors. As discussed by Harris and Hicks this procedure is justified by the little functional significance of the proximal area [20]. Furthermore, the longevity of this reduction by grinding is well documented by Zachrisson et al., which does not result in iatrogenic damage, such as gingival problems or dental caries [11]. In addition, regressive proportion directed the grinding remodeling of the supernumerary teeth.

On the other hand, scientific durability of adhesive system has been known [21, 22]. Thus, developing form, function, and natural aesthetics, it is promising with cosmetic remodeling by adding restorative material. Deep stains cannot be removed by enamel microabrasion, even, in association with an addition of a thin layer of restorative material as can be seen in left lateral incisor. Nevertheless, composite resin can be repolished or changed with little preparation of the tooth surface, therefore preserving tooth structure. In the case presented, a diagnostic mock-up was previously done to illustrate the possible conclusion of the cosmetic addition of resin and tissue condition of the gingival margin between the supernumerary teeth and left upper canine [16, 23].

Since the introduction of the Golden Proportion in dentistry by Lombardi [13] and Levin [12], the applications of this theory are numerous. Ricketts supported the use of these Divine Proportion ratios as guides for planning orthognathic surgery [24], while Furuse et al. suggest the Divine Proportion to harmonically allocate spaces between the anterior teeth for restorative treatment of multiple diastemata [25]. The Golden Proportion also can be used to guide dental remodeling by grinding and/or cosmetic remodeling by adding restorative material. The enamel grinding was performed to harmonically relate the successive width of the anterior teeth as viewed from the front aspect. At the same time, cosmetic remodeling by direct composite resin established new mesiodistal width for the maxillary central incisor.

Periodontal aspects before the restorative treatment are important and must be evaluated [26]. Oral instruction and prophylaxis were done before the restorative protocol. However, no periodontal surgery was done in the left superior central incisor because the gingival margin was not visible in her smile. Additionally, root coverage is achieved by many procedures like free gingival autografts. The patient had been previously submitted to a free gingival autograft in the lower right canine. Several studies state that root coverage using connective tissue grafts has high success rates. However, it also has disadvantage like less harmonic postoperative color, such as occurred with the patient presented in the lower right tooth in a previous surgical procedure. Other possibilities were discussed to solve the problem, but the patient preferred to avoid another surgical procedure.

Thus, the cosmetic remodeling teeth (by grinding or addicting material) can be a conservative and aesthetic alternative to reestablish the form, shape, and color. The association with techniques such as enamel microabrasion and dental bleaching is possible. And the Golden Proportion can be used to guide dental remodeling to improve the harmony of the smile and the facial composition.
